# Carbon-Coated CuNb_13_O_33_ as A New Anode Material for Lithium Storage

**DOI:** 10.3390/ma16051818

**Published:** 2023-02-22

**Authors:** Jiazhe Gao, Songjie Li, Wenze Wang, Yinjun Ou, Shangfu Gao, Xuehua Liu, Chunfu Lin

**Affiliations:** Institute of Materials for Energy and Environment, School of Materials Science and Engineering, Qingdao University, Qingdao 266071, China

**Keywords:** CuNb_13_O_33_, anode, in-situ XRD, unit-cell-volume change, electrochemical property

## Abstract

Niobates are very promising anode materials for Li^+^-storage rooted in their good safety and high capacities. However, the exploration of niobate anode materials is still insufficient. In this work, we explore ~1 wt% carbon-coated CuNb_13_O_33_ microparticles (C-CuNb_13_O_33_) with a stable shear ReO_3_ structure as a new anode material to store Li^+^. C-CuNb_13_O_33_ delivers a safe operation potential (~1.54 V), high reversible capacity of 244 mAh g^−1^, and high initial-cycle Coulombic efficiency of 90.4% at 0.1C. Its fast Li^+^ transport is systematically confirmed through galvanostatic intermittent titration technique and cyclic voltammetry, which reveal an ultra-high average Li^+^ diffusion coefficient (~5 × 10^–11^ cm^2^ s^−1^), significantly contributing to its excellent rate capability with capacity retention of 69.4%/59.9% at 10C/20C relative to 0.5C. An in-situ XRD test is performed to analyze crystal-structural evolutions of C-CuNb_13_O_33_ during lithiation/delithiation, demonstrating its intercalation-type Li^+^-storage mechanism with small unit-cell-volume variations, which results in its capacity retention of 86.2%/92.3% at 10C/20C after 3000 cycles. These comprehensively good electrochemical properties indicate that C-CuNb_13_O_33_ is a practical anode material for high-performance energy-storage applications.

## 1. Introduction

Compared with the traditional nickel-iron, nickel-metal, and lead-acid batteries, lithium-ion batteries (LIBs) are much more popular power sources for consumable electronics and electric vehicles due to the higher energy density, higher power density, and low self-discharge [1,2,3,4,5,6,7,8]. To satisfy the fast development need from electric vehicles, the exploration of higher-performance electrode materials is highly necessary [9]. At present, the most popular anode material is based on intercalation-type graphite due to its low cost and high practical capacity of 330–360 mAh g^−1^ [10,11]. However, it suffers a safety issue of lithium-dendrite formation when fast discharged/charged at its low potential plateau (<0.1 V) [12]. Intercalation-type Li_4_Ti_5_O_12_, which is the second most popular anode material, delivers a safe operation potential (~1.55 V), successfully avoiding the above safety issue [13]. Unfortunately, its insufficient practical capacity (~170 mAh g^−1^) limits its wide applications [14]. Hence, it is desirable to develop new anode materials with both large practical capacities and safe performance [15].

Recently, niobates with a high niobium valance of +5 have been served as high-performance anode materials [16,17,18,19]. The active Nb^4+^/Nb^5+^ and Nb^3+^/Nb^4+^ redox couples result in larger practical capacities than Li_4_Ti_5_O_12_ and safer operation potentials than graphite. Additionally, the large anion/cation ratios in niobates result in open crystal structures, enabling fast Li^+^ transport [20]. So far, a few niobates have been extensively studied as anode materials for Li^+^ storage. For instance, TiNb_2_O_7_ was firstly used as an anode material by Goodenough et al. in 2011, which showed a high reversible capacity of 285 mAh g^−1^ (0.1C) [21]. The Ti_2_Nb_10_O_29_ microparticles were synthesized by Wu et al., which exhibited a high reversible capacity of 247 mAh g^−1^ (0.1C) and high rate capability (130 mAh g^−1^ at 10C) [22]. The Ni_2_Nb_34_O_87_ microparticles were reported by Lv et al., which delivered high rate capability (capacity retention of 57.5% at 10C relative to 0.5C at 25 °C, 64.0% at 2C relative to 0.5C at −10 °C, and 65.3% at 10C relative to 0.5C at 60 °C) [23]. The Cu_2_Nb_34_O_87_ microparticles were synthesized by Yang et al., which presented a high reversible capacity of 343 mAh g^−1^ (0.1C), high rate capability (184 mAh g^−1^ at 10C) and good cyclability (88.5% capacity retention after 1000 cycles at 10C) [19]. However, the challenge remains to explore more niobate anode materials for LIBs.

Here, we explore CuNb_13_O_33_ with a stable ReO_3_ crystal structure as a new niobate anode material. To improve the electrical conduction among the CuNb_13_O_33_ microparticles, a carbon-coating strategy is employed. The carbon-coated CuNb_13_O_33_ (C-CuNb_13_O_33_) exhibits fast Li^+^ diffusivity with 5.01 × 10^–11^ cm^2^ s^−1^, significantly contributing to its outstanding rate capability (capacity percentage of 69.4%/59.9% at 10C/20C relative to 0.5C). Its maximum unit-cell-volume change is only 7.61%, leading to its superior cyclability (86.2%/92.3% capacity retention after 3000 cycles at 10C/20C). Furthermore, C-CuNb_13_O_33_ shows a high practical capacity (244 mAh g^−1^), high initial-cycle Coulombic efficiency (90.4%), and safe operation potential (~1.54 V) at 0.1C. These comprehensively good electrochemical properties of C-CuNb_13_O_33_ indicates that it is an ideal anode material of the LIBs for electric vehicles.

## 2. Materials and Methods

CuNb_13_O_33_ was prepared using solid-state reaction. 1 mmol Cu_2_O (Macklin, 97.0%) and 13 mmol Nb_2_O_5_ (AD 8758, Companhia Brasileira de Metalurgia e Mineração (CBMM)) were milled in a high-energy ball miller (SPEX 8000M, Metuchen, NJ, USA) for 1 h. After sintering the milled mixture at 900 °C for 4 h in Ar, CuNb_13_O_33_ microparticles were obtained. Then, 0.56 mmol CuNb_13_O_33_ and 0.35 mmol lactose (Macklin, 98.0%) were mixed in water under stirring at 80 °C until the mixture was fully dried. The obtained solid was heated at 700 °C for 2 h in Ar for carbonization, forming carbon-coated CuNb_13_O_33_ (C-CuNb_13_O_33_).

The X-ray diffraction (XRD) data of C-CuNb_13_O_33_ was recorded by an X-ray diffractometer (Rigaku Ultima IV, Tokyo, Japan) with Cu-K*α* radiation. The in-situ XRD test was performed by a specially-designed electrochemical cell module (Scistar LIB-XRD) equipped with a low-X-ray-absorption Be window [24]. The lattice-paraments were determined by Rietveld refinements of the powder and in-situ XRD patterns conducted on the General Structure Analysis System (GSAS) software (Revision 1253) with the EXPGUI interface [25]. The particle size, microstructure, and selected area electron diffraction (SAED) pattern were recorded by a field emission scanning electron microscopy (FESEM, JEOL JSM-7800F, Tokyo, Japan) equipped with energy-dispersive X-ray spectroscopy (EDX, OXFORD X-Max, Oxford, UK), and high-resolution transmission electron microscopy (HRTEM, JEOL JEM-2100, Tokyo, Japan). The weight percentage of carbon in C-CuNb_13_O_33_ was determined by a thermogravimetry analyzer (TGA, TGA 2, Mettler Toledo, Zurich, Switzerland). The elemental valence states of C-CuNb_13_O_33_ were examined by X-ray photoelectron spectroscopy (XPS, PHI5000 Versaprobe III, Mausaki, Japan).

The CR2032-type coin cells, which were fabricated in an Ar-filled glove box, were used to assess the electrochemical properties of C-CuNb_13_O_33_. C-CuNb_13_O_33_ (70 wt%), Super-P^®^ conductive carbon (20 wt%), and polyvinylidene fluoride (10 wt%) were mixed in N-methylpyrrolidone. After stirred for 8 h, the uniform slurry was casted on a Cu current collector, which was dried in a vacuum oven at 110 °C for 10 h. It was cut to circular electrodes with a diameter of 12 mm, obtaining the electrodes with active-material loadings of ~1.0 mg cm^−2^. The electrolyte was consisted of 1 M LiPF_6_ in an ethylene carbonate/diethylene carbonate/dimethyl carbonate mixed solvent (1:1:1 in volume). Glass fibers (Whatman GF/D-1823) were used as separators. The Li-metal foils were served as both counter and reference electrodes. Galvanostatic charge–discharge (GCD) tests were conducted on an automatic battery testing system (CT-3008, Neware, Shenzhen, China) at room temperature. An electrochemical workstation (Gamry Interface 1010E, Philadelphia, PA, USA) was employed to record the cyclic voltammogram (CV) profiles. All the electrochemical properties were examined within 1.0–3.0 V.

## 3. Results and Discussion

### 3.1. Physico–Chemical Characterizations

**Figure 1:**
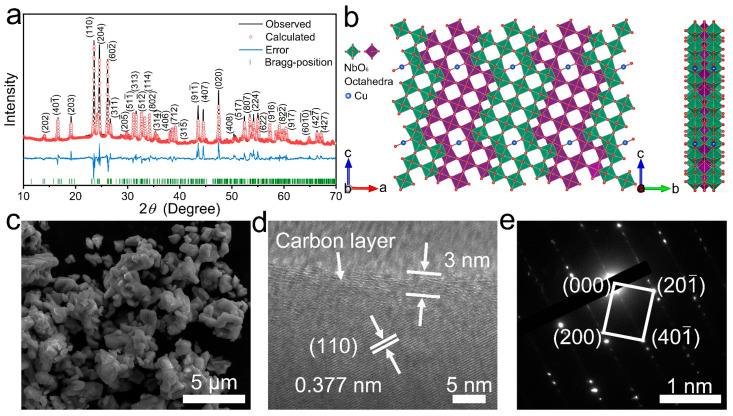
Physico–chemical characterizations of C-CuNb_13_O_33_. (**a**) XRD pattern of C-CuNb_13_O_33_ with Rietveld-refinement (main diffraction peaks are labeled). (**b**) Crystal structure of CuNb_13_O_33_ (*C2/m*). (**c**) FESEM image. (**d**) HRTEM image. (**e**) SAED pattern.

The Rietveld-refined XRD data (Figure 1a) indicates that CuNb_13_O_33_ has a monoclinic lattice with *C2/m* space group. The lattice parameters of CuNb_13_O_33_ are Rietveld-refined to be *a* = 22.49305(113) Å, *b* = 3.82579(17) Å, *c* = 15.40986(76) Å, *β* = 91.336(4)°, and *V* = 1325.714(140) Å^3^ (Table S1), and its fraction atomic parameters are listed in Table S2 (Supplementary Materials). The crystal structure of CuNb_13_O_33_ (Figure 1b) is constructed by NbO_6_ octahedra spreading on different shear *ac*-planes [26]. The NbO_6_ octahedra are connected by sharing corners and edges in each layer. However, the arrangement is interrupted by Cu^+^ in a regular way. Each Cu^+^ is connected to two O^2^-ions and occupies the 2*c* site. Moreover, the interlayers share octahedron edges. This special octahedron connection results in a stable A–B–A layered structure. The resulting tunnels in this crystal structure are beneficial for Li^+^ transport and storage. Figure S1 shows the XPS spectra of Nb and Cu elements in C-CuNb_13_O_33_. The Nb-3*d* spectrum consists of a Nb-3*d*_5/2_ and Nb-3*d*_3/2_ doublet respectively at 207.8 and 210.5 eV (Figure S1a) [24], and the Cu-2*p* spectrum comprises a Cu-2*p*_3/2_ and Cu-2*p*_1/2_ doublet respectively at 932.5 and 952.5 eV (Figure S1b) [27], indicating that the valences states of Nb and Cu elements are respectively +5 and +1, as expected.

The FESEM image (Figure 1c) of C-CuNb_13_O_33_ exhibits that its particle size distributes in a range of 1–5 μm. Its HRTEM image (Figure 1d) indicates that the *d*-spacing of 0.377 nm is indexed to the (110) crystallographic plane and that the carbon layer has a thickness of ~3 nm. The carbon content of C-CuNb_13_O_33_ is calculated to be ~1 wt% from its TGA result (Figure S2). The regular diffraction spots from CuNb_13_O_33_ are shown in the SAED pattern (Figure 1e), matching well with its (200), (20$\bar{1}$), and (40$\bar{1}$) crystallographic planes, which confirms its monoclinic structure with *C2/m* space group. The EDX mapping images (Figure S3) show uniform C, Cu, Nb, and O elements distributions with only tiny Cu precipitation.

### 3.2. Li^+^-Storage Properties

**Figure 2:**
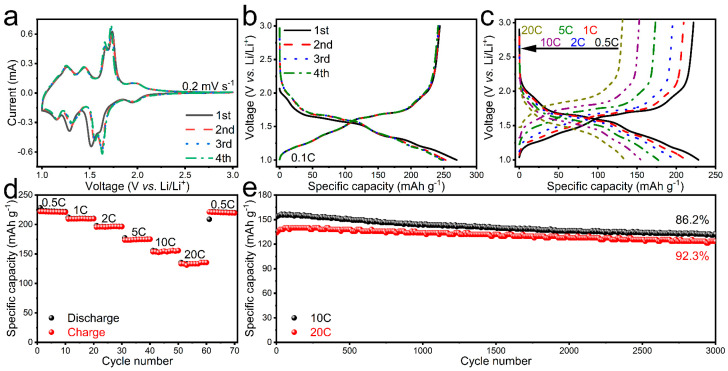
Electrochemical properties of C-CuNb_13_O_33_/Li half-cell. (**a**) CV curves at 0.2 mV s^−1^. (**b**) Initial four-cycle GCD profiles at 0.1C. (**c**) GCD profiles at different current rates, (**d**) rate capability, (**e**) Cyclability over 3000 cycles at 10C and 20C. 1C = 378 mAh g^−1^.

To study the redox mechanism of C-CuNb_13_O_33_, a CV experiment is performed on the C-CuNb_13_O_33_/Li half-cell (Figure 2a). The initial cycle is different from the following one, which can be attributed to the irreversible polarization and the formation of thin SEI films [28]. However, the CV profiles show good repeatability after the initial cycle. The second cycle exist four obvious peak pairs respectively located at 1.63/1.73, 1.55/1.67, 1.30/1.44 and 1.16/1.26 V. The first and second pairs could be ascribed to the Nb^4+^/Nb^5+^ redox reaction, while the third and fourth pairs could correspond to the Nb^3+^/Nb^4+^ redox reaction [29].

Figure 2b shows the GCD profiles of the C-CuNb_13_O_33_/Li half-cell within 1.0–3.0 V at 0.1C. C-CuNb_13_O_33_ exhibits a large first-cycle discharge/charge capacity (270/244 mAh g^−1^) and high Coulombic efficiency (i.e., the charge capacity divided by the discharge capacity is 90.4%). The average operation potential during lithiation/delithiation is ~1.54 V, which is similar to the popular Li_4_Ti_5_O_12_ and indicates the high safety performance of C-CuNb_13_O_33_. With increasing the current rate, C-CuNb_13_O_33_ is capable of retaining large reversible capacities of 222, 209, 195, 174, 154, and 133 mAh g^−1^ for ten cycles each at 0.5C, 1C, 2C, 5C, 10C, and 20C, respectively (Figure 2c,d), revealing its excellent rate capability with capacity percentage of 69.4%/59.9% at 10C/20C relative to 0.5C. Meanwhile, the capacity has no obvious decay when the rate returns from 20C to 0.5C. Long-term discharge/charge tests are further performed at 10C and 20C, revealing superior cyclability with 86.2% and 92.3% retention after 3000 cycles, respectively (Figure 2e). In addition, the comprehensive properties of C-CuNb_13_O_33_ are better than not only those of CuNb_13_O_33_ (Figure S4) but also those of most intercalation-type anode materials previously reported (Table S3).

### 3.3. Electrochemical Kinetics

**Figure 3:**
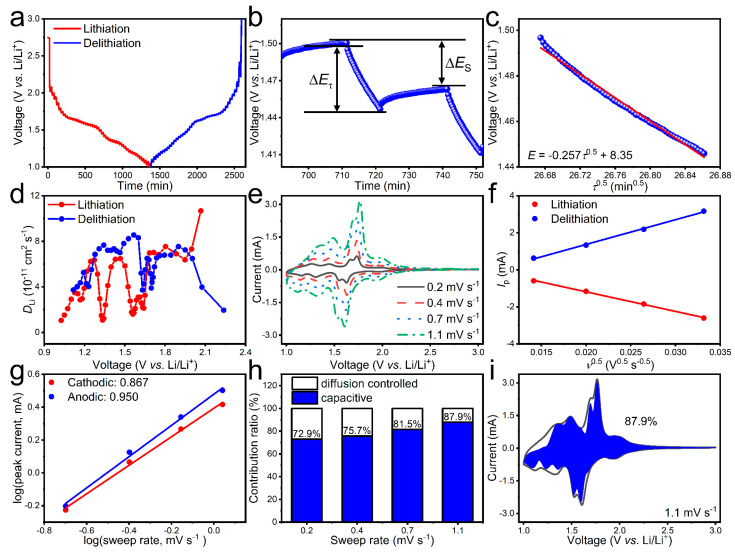
Electrochemical kinetics of C-CuNb_13_O_33_/Li half-cell. (**a**) GITT lithiation/delithiation profile. (**b**) Potential (*E*) and time (*t*) profile of single step in GITT test. (**c**) Relationship between *E* and square root of titration duration (*τ*^0^.^5^) during a typical titration. (**d**) Variations of $D_{\mathrm{Li}}^{\mathrm{GITT}}$ during lithiation/delithiation. (**e**) CV curves at variable sweep rates. (**f**) Relationship between peak current (*I*_p_) and square root of sweep rate (*ν*^0^.^5^) for intensive cathodic/anodic peaks. (**g**) Calculations of *b* values through the relationship between *I*_p_ and *ν*. (**h**) Pseudocapacitive contribution ratios at different sweep rates. (**i**) Pseudocapacitive contribution ratio at 1.1 mV s^−1^.

To study the Li^+^ transport kinetics of C-CuNb_13_O_33_, the Li^+^ apparent diffusion coefficients (*D*_Li_) during lithiation/delithiation are determined based on both the GITT and CV techniques. Figure 3a shows the GITT profiles of the C-CuNb_13_O_33_/Li half-cell during the initial cycle at 0.1C. The $D_{\mathrm{Li}}^{\mathrm{GITT}}$ value can be calculated by using Equation (1), which is rooted from Fick’s second law [30]:

(1) $$D_{\mathrm{Li}}^{\mathrm{GITT}}=\frac{4}{\pi }{\left(\frac{m_{a}V_{m}}{M_{a}S}\right)}^{2}{\left(\frac{\Delta E_{s}}{\tau \left({dE}_{\tau }/d\sqrt{\tau }\right)}\right)}^{2}\;\left(\tau \;\ll \;\frac{L^{2}}{D_{\mathrm{Li}}^{\mathrm{GITT}}}\right)$$

where *m*_a_, *M*_a_, *V*_m_, *S*, and *L* present the mass, molar mass, molar volume, electrode surface area, and electrode thickness of C-CuNb_13_O_33_, respectively; *τ* is the time during which a constant current is applied; Δ*E*_s_ means the variation of the equilibrium potential, and Δ*E*_τ_ means the variation of potential during the current pulse, which can be gained from the GITT profiles (Figure 3b). Since a linear relationship is achieved between the potential *E* and *τ*^0^.^5^ during each titration (Figure 3c), Equation (1) can be simplified as Equation (2):

(2) $$D_{\mathrm{Li}}^{\mathrm{GITT}}=\frac{4}{\pi \tau }{\left(\frac{m_{b}V_{m}}{M_{b}S}\right)}^{2}{\left(\frac{\Delta E_{s}}{\Delta E_{\tau }}\right)}^{2}\;\left(\tau \;\ll \;\frac{L^{2}}{D_{\mathrm{Li}}^{\mathrm{GITT}}}\right)$$

Based on Equation (2), the $D_{\mathrm{Li}}^{\mathrm{GITT}}$ value of C-CuNb_13_O_33_ is determined to be 4.07 × 10^−11^ cm^2^ s^−1^/5.94 × 10^−11^ cm^2^ s^−1^ during lithiation/delithiation (Figure 3d).

The *D*_Li_ values of C-CuNb_13_O_33_ during lithiation/delithiation are also determined from its CV data at different sweep rates (Figure 3e). It is found that the *I*_p_ of the intensive cathodic/anodic reaction is in proportional to *v*^0^.^5^ (Figure 3f), which exhibits linear semi-infinite diffusion during lithiation/delithiation. Hence, the $D_{\mathrm{Li}}^{\mathrm{CV}}$ values can be determined based on the Randles–Servick equation Equation (3) [30]:

(3) $$I_{P}=0.4463\mathrm{nFAC}{\left(\frac{{\mathrm{nFvD}}_{\mathrm{Li}}^{\mathrm{CV}}}{\mathrm{RT}}\right)}^{0.5}$$

where *n*, *A*, and *C* mean the number of charge transfer, electrode area, and molar concentration of Li^+^ in solid, respectively, and *I*_p_, *F*, *v*, *R*, and *T* represent the peak current, Faraday constant, sweep rate, temperature, and gas constant, respectively. Equation (3) can be simplified as Equation (4) due to the fact that the electrochemical system is performed at 25 °C.

(4) $$I_{P}{=268,600n}^{1.5}{\mathrm{AD}}_{\mathrm{Li}}^{\mathrm{CV}}{}^{0.5}{\mathrm{Cv}}^{0.5}$$

Based on Equation (4), the $D_{\mathrm{Li}}^{\mathrm{CV}}$ value of C-CuNb_13_O_33_ is determined to be 2.85 × 10^−11^ cm^2^ s^−1^/4.55 × 10^−11^ during lithiation/delithiation, which matches with the GITT results. It should be emphasized that the average *D*_Li_ values of C-CuNb_13_O_33_ surpass those of the previously-reported niobates (Table S4). This fast Li^+^ diffusivity of C-CuNb_13_O_33_ is beneficial for achieving its excellent rate capability.

To analyze the capacitive behavior of C-CuNb_13_O_33_, the electrochemical kinetics obtained from the CV data (Figure 3e) is further investigated through the relationship between *I*_p_ and *ν* [31,32]:

(5) $$I_{p}{=\mathrm{av}}^{b}$$

where *a* and *b* are changeable parameters. *b* = 1 indicates 100% capacitive behavior, while *b* = 0.5 indicates 100% diffusion-controlled behavior [31]. To calculate the *b* value, Equation (5) can be transformed into Equation (6):

(6) $${\log I}_{p}=\log\;a+b\;\log\;v$$

The *b* values of C-CuNb_13_O_33_ for the cathodic and anodic peaks are respectively determined to be 0.867 and 0.950 (Figure 3g). These large *b* values indicate that the electrochemical process is dominated by capacitive control.

For further understanding the electrochemical kinetics of C-CuNb_13_O_33_, the capacitive contribution (*jv*) and the diffusion-controlled contribution (*kv*^1/2^) are determined based on Equation (7) [32]:

(7) $${I=\mathrm{jv}+\mathrm{kv}}^{1/2}$$

where *I* represents the detected current at a fixed potential, and *j* and *k* are changeable parameters. Equation (7) can be transformed to Equation (8). As a result, the *j* value is the slope obtained through the linear relationship between the *I*/*v*^1/2^ and *v*^1/2^, and thus the capacitive contribution ratio can be easily obtained.

(8) $${I/v}^{1/2}{=\mathrm{jv}}^{1/2}+k$$

The large capacitive contribution ratios of C-CuNb_13_O_33_ indicate dominant capacitive contributions at different sweep rates (Figure 3h), matching with the *b* values. It is worth noting that the capacitive contribution ratio of C-CuNb_13_O_33_ reaches 87.9% at 1.1 mV s^−1^ (Figure 3i). This significant capacitive behavior is also beneficial for the rate capability.

### 3.4. Crystal Structure Evolutions

**Figure 4:**
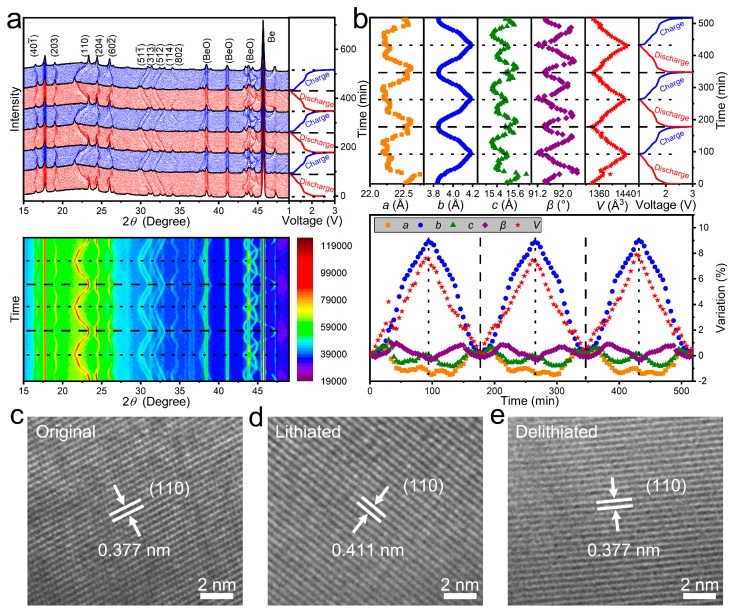
Crystal structure evolutions of C-CuNb_13_O_33_. (**a**) Original in-situ XRD patterns with GCD profiles (0.4C) and contour in-situ XRD patterns of C-CuNb_13_O_33_/Li in-situ cell (first three cycles). (**b**) Lattice-parameter variations of C-CuNb_13_O_33_ (initial three cycles). Ex-situ HRTEM characterization of (**c**) original, (**d**) lithiated (1.0 V), and (**e**) delithiated (3.0 V) C-CuNb_13_O_33_ samples.

An in-situ XRD test is performed to clarify crystal-structural evolutions of CuNb_13_O_33_ during lithiation/delithiation. Figure 4a exhibits the initial-three-cycle original and contour in-situ XRD patterns with GCD profiles. During the initial lithiation, all the peaks exhibit decreased intensities because the Li^+^ insertion undoubtedly decreases the lattice order [33]. The (110), (51$\bar{1}$), (313), (51$\bar{2}$), and (114) peaks shift toward smaller angles. The (204) and (60$\bar{2}$) peaks shift toward smaller angles until ~1.6 V and then slowly shifts towards larger angles until ~1.4 V. The (204) peak shifts toward smaller angles and the (60$\bar{2}$) peak continues shifting toward larger angles until ~1.0 V. Although these peak evolutions are complex, they almost fully recover their initial positions and intensities during the following delithiation. The subsequent peak evolutions, however, are almost similar to those in the initial cycle, indicating the excellent electrochemical stability of C-CuNb_13_O_33_. The lithiation/delithiation mechanism in CuNb_13_O_33_ can be described based on Equation (9):

CuNb_13_O_33_ + *x*e^−^ + *x*Li^+^ ↔ Li*_x_*CuNb_13_O_33_ (0 ≤ *x* ≤ 26) (9)

Figure 4b illustrates the lattice-parameter changes of C-CuNb_13_O_33_ gained by Rietveld-refining the in-situ XRD patterns. During the Li^+^ insertion, the *a* and *c*-value changes follow a sequence of increase → decrease → slight increase. The total *a*- and *c*-value changes are only −1.49 and −0.13%, respectively. The *b* value gradually increases with a maximum change of 8.93%. The obvious increase of the *b* value suggests that Li^+^ stores in the (010) crystallographic plane during lithiation. The *β* value first increases and then decreases with a total change of −0.33%. Consequently, the *V* value increases by 7.61%. During the Li^+^ extraction, all the lattice-parameter changes are very reversible to those during the Li^+^ insertion, which matches well with the peak evolutions. These changes of the lattice parameters are in good agreement with the ex-situ HRTEM result, which exhibits that the (110) *d*-spacing increases from 0.377 nm (pristine state, Figure 4c) to 0.411 nm (discharge to 1.0 V, Figure 4d) and returns to 0.377 nm (charge to 3.0 V, Figure 4e).

## 4. Conclusions

~1 wt% carbon-coated CuNb_13_O_33_ microparticles (C-CuNb_13_O_33_) with a stable shear ReO_3_ structure are explored as a new anode material with comprehensively good Li^+^ storage properties. This new material owns the Nb^4+^/Nb^5+^ and Nb^3+^/Nb^4+^ redox couples, enabling a safe operation potential (~1.54 V), high practical capacity (244 mAh g^−1^), and a high initial-cycle Coulombic efficiency (90.4%) at 0.1C. Its very fast Li^+^ transport (5.01 × 10^−11^ cm^2^ s^−1^) and significant capacitive behavior result in its excellent rate capability with capacity percentages of 69.4% (10C relative to 0.5C) and 59.9% (20C relative to 0.5C). Additionally, it exhibits superior cyclability with capacity retention of 86.2%/92.3% at 10C/20C after 3000 cycles due to its maximum unit-cell-volume change of only 7.61%. Therefore, C-CuNb_13_O_33_ is an ideal anode material for Li^+^-storage.

## Data Availability

The data presented in this study are available on request from the corresponding author. The data are not publicly available due to privacy.

## Supplementary Materials

The following supporting information can be downloaded at: https://www.mdpi.com/article/10.3390/ma16051818/s1, Figure S1: XPS spectra of Nb and Cu elements in C-CuNb13O33; Figure S2: TGA curves of CuNb13O33 and C-CuNb13O33.; Figure S3: EDX mapping images of C-CuNb13O33.; Figure S4: Electrochemical properties of CuNb13O33/Li half-cell; Table S1: Details of Rietveld-refinement and crystal data of CuNb13O33; Table S2: Fractional atomic parameters of CuNb13O33 with C2/m; Table S3: Comparisons of electrochemical properties of C-CuNb13O33 with those of intercalation-type anode materials previously reported; Table S4: Comparisons of apparent Li^+^ diffusion coefficient (*D*_Li_) of C-CuNb_13_O_33_ with that of previously-reported niobates at 25 °C. References [19,23,24,28,34,35,36,37,38,39,40,41,42,43,44,45,46,47,48,49] are cited in the supplementary materials.
