# Supplementary material for: Carbon-Coated CuNb13O33 as A New Anode Material for Lithium Storage
Source: Materials (Basel). 2023 Feb 22;16(5):1818. doi: 10.3390/ma16051818 (PMC10004636; doi:10.3390/ma16051818)
Supplement: Supplementary file 1 [file materials-16-01818-s001.zip › materials-2179022-supplementary.pdf]

Supporting Information

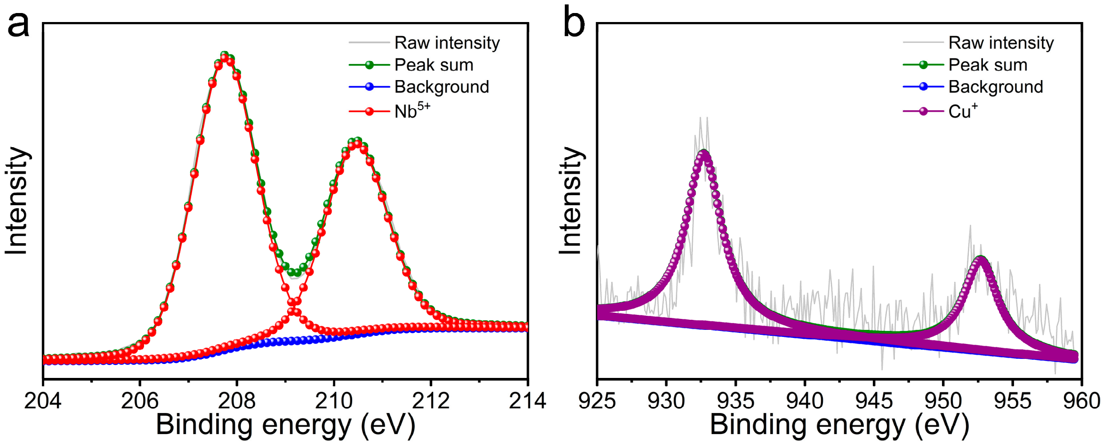

Figure S1. XPS spectra of a) Nb and b) Cu elements in C-$CuNb_{13}O_{33}$.

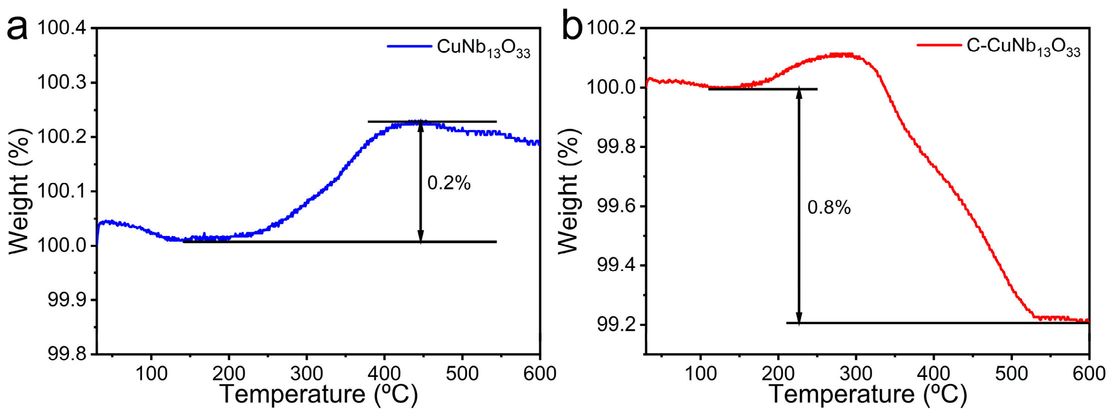

Figure S2. TGA curves of (a) $CuNb_{13}O_{33}$ and (b) C-$CuNb_{13}O_{33}$.

Calculation of coated-carbon weight percentage:

Figure S2a indicates that the weight of $CuNb_{13}O_{33}$ increases by ~0.2 wt% at the high temperature due to the Cu^+ oxidation. **Figure S2b** shows that the weight decrease (~0.8 wt%) of C-$CuNb_{13}O_{33}$ can be attributed to the combination of the Cu^+ oxidation and carbon volatilization. Therefore, the carbon weight percentage of C-$CuNb_{13}O_{33}$ can be calculated to be ~1 wt%.

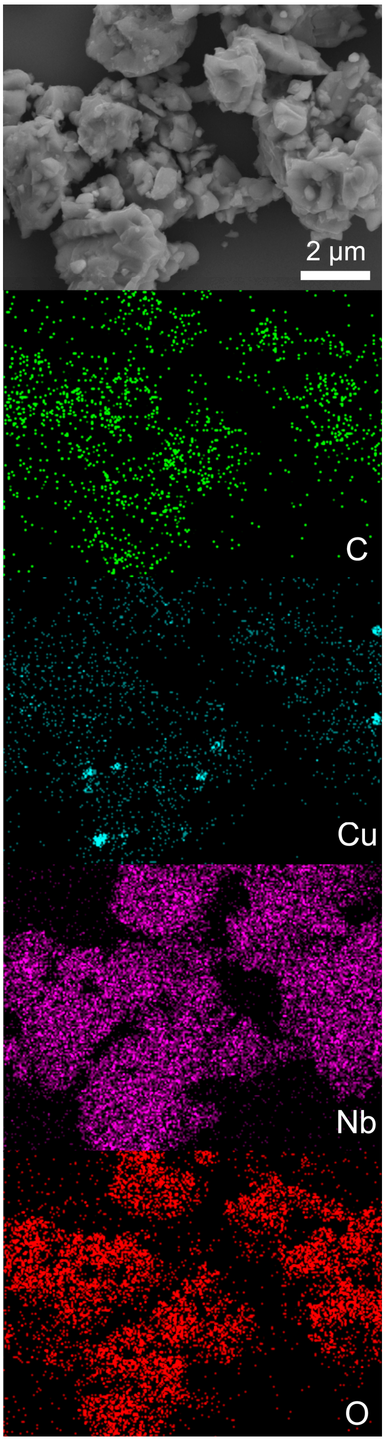

Figure S3. EDX mapping images of C-$CuNb_{13}O_{33}$.

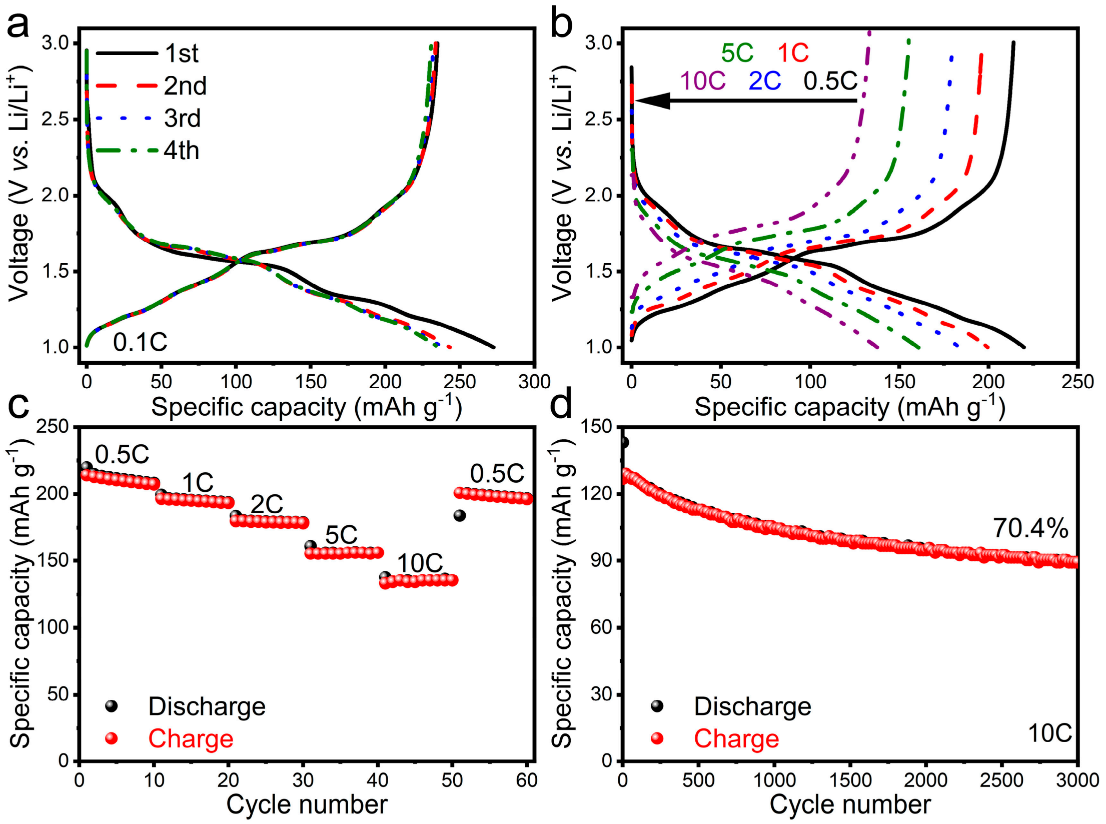

Figure S4. Electrochemical properties of $CuNb_{13}O_{33}$/Li half cell. (a) Initial four-cycle GCD profiles at 0.1C. (b) GCD profiles at different current rates, (c) Rate capability, (d) Cyclability over 3000 cycles at 10C.

For comparison, the electrochemical properties of $CuNb_{13}O_{33}$ were tested by the same way with C-$CuNb_{13}O_{33}$. The GCD profiles of the $CuNb_{13}O_{33}$/Li half cell at 0.1C (**Figure S4a**) exhibits a first-cycle discharge/charge capacity (273/235 mAh g^{-1}) and Coulombic efficiency (86.1%). When the current rate is increased to 0.5C, 1C, 2C, 5C, and 10C, $CuNb_{13}O_{33}$ retains reversible capacities of 214, 196, 180, 155, and 133 mAh g^{-1}, respectively (**Figure S4b** and **Figure S4c**), revealing its insufficient rate capability with a 10C *vs*. 0.5C capacity ratio of 62.1%. Furthermore, $CuNb_{13}O_{33}$ delivers insufficient cyclability with capacity retention of 70.4% at 10C over 3000 cycles

(**Figure S4d**). Clearly, the comprehensive properties of $CuNb_{13}O_{33}$ are inferior to those of C-$CuNb_{13}O_{33}$.

Table S1. Details of Rietveld refinement and crystal data of $CuNb_{13}O_{33}$.

Sample	Microparticles
Diffractometer	Rigaku Ultima IV
Radiation type	Cu-Kα
Wavelength (Å)	1.5418
Refined profile range (°2θ)	10–70
Step size (°2θ)	0.02
R_p	6.61%
R_{wp}	9.36%
Formula	$CuNb_{13}O_{33}$
Symmetry	Monoclinic
Space group	*C2/m*
a (Å)	22.49305(113)
b (Å)	3.82579(17)
c (Å)	15.40986(76)
β (°)	91.336(4)
Volume (Å^3)	1325.714(140)
Z	2

Table S2. Fractional atomic parameters of $CuNb_{13}O_{33}$ with *C2/m*.

atom	site	symmetry	*x*	*y*	*z*	occupancy
Cu	2*c*	*2/m*	0.5	0.5	0.5	1
Nb1	2*a*	*2/m*	0	0	0	1
Nb2	4*i*	*m*	0.071938	0	0.232478	1
Nb3	4*i*	*m*	0.222077	0	0.136094	1
Nb4	4*i*	*m*	0.154666	0	0.907204	1
Nb5	4*i*	*m*	0.139552	0	0.467690	1
Nb6	4*i*	*m*	0.296140	0	0.374660	1
Nb7	4*i*	*m*	0.091678	0	0.671544	1
O1	2*b*	*2/m*	0	0.5	0	1
O2	4*i*	*m*	0.082733	0	0.946845	1
O3	4*i*	*m*	0.386091	0	0.201279	1
O4	4*i*	*m*	0.250532	0	0.844722	1
O5	4*i*	*m*	0.158853	0	0.192071	1
O6	4*i*	*m*	0.467637	0	0.746295	1
O7	4*i*	*m*	0.186116	0	0.037983	1
O8	4*i*	*m*	0.037918	0	0.126731	1
O9	4*i*	*m*	0.136734	0	0.783042	1
O10	4*i*	*m*	0.288429	0	0.251315	1
O11	4*i*	*m*	0.104930	0	0.358436	1
O12	4*i*	*m*	0.012828	0	0.708890	1
O13	4*i*	*m*	0.396345	0	0.332870	1
O14	4*i*	*m*	0.207187	0	0.673700	1
O15	4*i*	*m*	0.233263	0	0.447916	1
O16	4*i*	*m*	0.338730	0	0.523545	1
O17	4*i*	*m*	0.063227	0	0.552625	1

Table S3. Comparisons of electrochemical properties of C-$CuNb_{13}O_{33}$ with those of intercalation-type anode materials previously reported.

material	reversible capacity at 0.1C	rate capability	cyclability	reference
**C-$CuNb_{13}O_{33}$**	**244 mAh g^{-1}**	**155/133 mAh g^{-1} at 10/20C**	**86.2%/92.3% after 3,000 cycles at 10/20C**	**this work**
$TiNb_2O_7$	292 mAh g^{-1}	90 mAh g^{-1} at 10C	59.8% after 1,000 cycles at 10C	1
$Ti_2Nb_{10}O_{29}$	290 mAh g^{-1}	80 mAh g^{-1} at 10C	75.0% after 500 cycles at 10C	2
$TiNb_{24}O_{62}$	258 mAh g^{-1}	147 mAh g^{-1} at 10C	80.9% after 500 cycles at 10C	3
$MoNb_{12}O_{33}$	294 mAh g^{-1}	138 mAh g^{-1} at 10C	89.2% after 1,000 cycles at 10C	4
$FeNb_{11}O_{29}$	251 mAh g^{-1}	57 mAh g^{-1} at 10C	41.6% after 500 cycles at 10C	5
$GaNb_{11}O_{29}$	255 mAh g^{-1}	121 mAh g^{-1} at 10C	66.9% after 1,000 cycles at 10C	6
$AlNb_{11}O_{29}$	266 mAh g^{-1}	131 mAh g^{-1} at 10C	93.2% after 500 cycles at 10C	7
$Mg_2Nb_{34}O_{87}$	290 mAh g^{-1}	149 mAh g^{-1} at 10C	93.5% after 500 cycles at 10C	8
$Cu_2Nb_{34}O_{87}$	343 mAh g^{-1}	184 mAh g^{-1} at 10C	88.5% after 1,000 cycles at 10C	9
$CrNb_{11}O_{29}$	286 mAh g^{-1}	150 mAh g^{-1} at 10C	90.2% after 400 cycles at 10C	10
$Mo_3Nb_{14}O_{44}$	323 mAh g^{-1}	123 mAh g^{-1} at 10C	71.8% after 1,000 cycles at 10C	11
$Li_4Ti_5O_{12}$	162 mAh g^{-1}	90 mAh g^{-1} at 10C	89.3% after 5,000 cycles at 10C	12
Graphite	359 mAh g^{-1}	68 mAh g^{-1} at 10C	36.7% after 1,000 cycles at 10C	13

Table S4. Comparisons of apparent Li^+ diffusion coefficient (D_{Li}) of C-$CuNb_{13}O_{33}$ with that of previously-reported niobates at 25 °C.

material	D_{Li} ($cm^2\ s^{-1}$)	test technique	reference
**C-$CuNb_{13}O_{33}$ microparticles**	**5.01×10^{-11}**	**GITT**	**this work**
**C-$CuNb_{13}O_{33}$ microparticles**	**3.70×10^{-11}**	**CV**	**this work**
$MoNb_{12}O_{33}$ microparticles	3.9×10^{-14}	GITT	4
$Al_{0.5}Nb_{24.5}O_{62}$ microparticles	2.5×10^{-13}	GITT	14
$W_5Nb_{16}O_{55}$ microparticles	1.0×10^{-13}	GITT	14
$Cu_2Nb_{34}O_{87}$ microparticles	3.5×10^{-13}	GITT	9
VNb_9O_{25} nanoribbons	5.17×10^{-15}	EIS	15
$TiNb_6O_{17}$ microparticles	4.88×10^{-14}	CV	16
$Ti_2Nb_{10}O_{27.1}$ microparticles	2.11×10^{-14}	CV	17
$Cu_{0.02}Ti_{0.94}Nb_{2.04}O_7$ microparticles	1.66×10^{-14}	EIS	1
$TiNb_2O_7$ microparticles	1.05×10^{-15}	EIS	1
$Nb_{25}O_{62}$ microparticles	6.75×10^{-14}	EIS	18
$Nb_{12}O_{29}$ microparticles	5.42×10^{-15}	EIS	18
$TiCr_{0.5}Nb_{10.5}O_{29}$ nano-sized particles	2.07×10^{-14}	CV	19
$Cr_{0.5}Nb_{24.5}O_{62}$ microparticles	4.57×10^{-14}	EIS	20
$Ti_2Nb_{10}O_{29}$ microparticles	1.55×10^{-15}	EIS	2

References

[1] C. Yang, C. Lin, S. Lin, Y. Chen, J. Li, $Cu_{0.02}Ti_{0.94}Nb_{2.04}O_7$: an advanced anode material for lithium-ion batteries of electric vehicles, J. Power Sources 328 (2016) 336–344.

[2] C. Yang, S. Yu, Y. Ma, C. Lin, Z. Xu, H. Zhao, S. Wu, P. Zheng, Z.Z. Zhu, J. Li, N. Wang, Cr^{3+} and Nb^{5+} co-doped $Ti_2Nb_{10}O_{29}$ materials for high-performance lithium-ion storage, J. Power Sources 360 (2017) 470–479.

[3] C. Yang, S. Deng, C. Lin, S. Lin, Y. Chen, J. Li, H. Wu, Porous $TiNb_{24}O_{62}$ microspheres as high-performance anode materials for lithium-ion batteries of electric vehicles, Nanoscale 8 (2016) 18792–18799.

[4] X. Zhu, J. Xu, Y. Luo, Q. Fu, G. Liang, L. Luo, Y. Chen, C. Lin, X. Zhao, $MoNb_{12}O_{33}$ as a new anode material for high-capacity, safe, rapid and durable Li^+ storage: structural characteristics, electrochemical properties and working mechanisms, J. Mater. Chem. A 7 (2019) 6522–6532.

[5] X. Lou, Z. Xu, Z. Luo, C. Lin, C. Yang, H. Zhao, P. Zheng, J. Li, N. Wang, Y. Chen, H. Wu, Exploration of $Cr_{0.2}Fe_{0.8}Nb_{11}O_{29}$ as an advanced anode material for lithium-ion batteries of electric vehicles, Electrochim. Acta 245 (2017) 474–480.

[6] X. Lou, Q. Fu, J. Xu, X. Liu, C. Lin, J. Han, Y. Luo, Y. Chen, X. Fan, J. Li, $GaNb_{11}O_{29}$ nanowebs as high-performance anode materials for lithium-ion batteries, ACS Appl. Nano Mater. 1 (2018) 183–190.

[7] X. Lou, R. Li, X. Zhu, L. Luo, Y. Chen, C. Lin, H. Li, X. Zhao, New anode material for lithium-ion batteries: aluminum niobate ($AlNb_{11}O_{29}$), ACS Appl. Mater. Interfaces

11 (2019) 6089–6096.

[8] X. Zhu, Q. Fu, L. Tang, C. Lin, J. Xu, G. Liang, R. Li, L. Luo, Y. Chen, $Mg_2Nb_{34}O_{87}$ porous microspheres for use in high-energy, safe, fast-charging, and stable lithium-ion batteries, ACS Appl. Mater. Interfaces 10 (2018) 23711–23720.

[9] L. Yang, X. Zhu, X. Li, X. Zhao, K. Pei, W. You, X. Li, Y. Chen, C. Lin, R. Che, Conductive copper niobate: superior Li^+-storage capability and novel Li^+-transport mechanism, Adv. Energy Mater. 9 (2019) 1920174.

[10] Q. Fu, X. Liu, J. Hou, Y. Pu, C. Lin, L. Yang, X. Zhu, L. Hu, S. Lin, L. Luo, Y. Chen, Highly conductive $CrNb_{11}O_{29}$ nanorods for use in high-energy, safe, fast-charging and stable lithium-ion batteries, J. Power Sources 397 (2018) 231–239.

[11] R. Li, G. Liang, X. Zhu, Q. Fu, Y. Chen, L. Luo, C. Lin, $Mo_3Nb_{14}O_{44}$: a new Li^+ container for high-performance electrochemical energy storage, Energy Environ. Mater. 4 (2021) 65–71.

[12] W. Wang, Q. Zhang, T. Jiang, S. Li, J. Gao, X. Liu, C. Lin, Conductive $LaCeNb_6O_{18}$ with a very open A-site-cation-deficient perovskite structure: a fast- and stable-charging Li^+-storage anode compound in a wide temperature range, Adv. Energy Mater. 12 (2022) 2200656.

[13] C. Lv, C. Lin, X. Zhao, Rational design and synthesis of nickel niobium oxide with high-rate capability and cycling stability in a wide temperature range, Adv. Energy Mater. 12 (2022) 2102550.

[14] Q. Fu, R. Li, X. Zhu, G. Liang, L. Luo, Y. Chen, C. Lin, X. Zhao, Design, synthesis and lithium-ion storage capability of $Al_{0.5}Nb_{24.5}O_{62}$, J. Mater. Chem. A 7 (2019) 19862–

19871.

[15] S. Qian, H. Yu, L. Yang, H. Zhu, X. Cheng, Y. Xie, N. Long, M. Shui, J. Shu, High-rate long-life pored nanoribbon VNb_9O_{25} built by interconnected ultrafine nanoparticles as anode for lithium-ion batteries, ACS Appl. Mater. Interfaces 9 (2017) 30608–30616.

[16] C. Lin, G. Wang, S. Lin, J. Li, L. Lu, $TiNb_6O_{17}$: a new electrode material for lithium-ion batteries, Chem. Commun. 51 (2015) 8970–8973.

[17] C. Lin, S. Yu, H. Zhao, S. Wu, G. Wang, L. Yu, Y. Li, Z. Zhu, J. Li, S. Lin, Defective $Ti_2Nb_{10}O_{27.1}$: an advanced anode material for lithium-ion batteries, Sci. Rep. 5 (2015) 17836.

[18] R. Li, Y. Qin, X. Liu, L. Yang, C. Lin, R. Xia, S. Lin, Y. Chen, J. Li, Conductive $Nb_{25}O_{62}$ and $Nb_{12}O_{29}$ anode materials for use in high-performance lithium-ion storage, Electrochim. Acta 266 (2018) 202–211.

[19] L. Hu, R. Lu, L. Tang, R. Xia, C. Lin, Z. Luo, Y. Chen, J. Li, $TiCr_{0.5}Nb_{10.5}O_{29}$/CNTs nanocomposite as an advanced anode material for high-performance Li^+-ion storage, J. Alloys Compd. 732 (2018) 116–123.

[20] C. Yang, S. Yu, C. Lin, F. Lv, S. Wu, Y. Yang, W. Wang, Z. Zhu, J. Li, N. Wang, S. Guo, $Cr_{0.5}Nb_{24.5}O_{62}$ nanowires with high electronic conductivity for high-rate and long-life lithium-ion storage, ACS Nano 11 (2017) 4217–4224.
